# Prevention of adverse psychological effects and social stigma during COVID-19 pandemic: Solutions

**DOI:** 10.17179/excli2021-3414

**Published:** 2021-02-10

**Authors:** Ahmad Soltani, Mahmoudreza Peyravi, Mohammad Hossein Taghrir, Mahnaz Ahmadi, Mahnaz Dehbozorgi, Faegheh Jomeh Pour, Rita Rezaee, Milad Ahmadi Marzaleh

**Affiliations:** 1 Research Center for Emergency and Disaster Resilience, Red Crescent Society of the Islamic Republic of Iran, Tehran, Iran; 2 Iran-Helal Institute of Applied-Science and Technology, Red Crescent Society of the Islamic Republic of Iran, Tehran, Iran; 3 Department of Health in Disasters and Emergencies, School of Management and Medical Informatics, Shiraz University of Medical Sciences, Shiraz, Iran; 4 Student Research Committee, School of Medicine, Shiraz University of Medical Sciences, Shiraz, Iran; 5 Clinical Research Development Center, Imam Reza Hospital, Kermanshah University of Medical Sciences, Kermanshah, Iran; 6 Woman and Family Studies, E-Campus, Islamic Azad University, Tehran, Iran; 7 Obstetrics and Gynecology, Hormozgan University of Medical Sciences, Bandar Abbas, Iran; 8 Department of Health Information Management, Health Human Resources Research Center, School of Management and Medical Informatics, Shiraz University of Medical Sciences, Shiraz, Iran; 9 Research Center for Health Management in Mass Gathering, Red Crescent Society of the Islamic Republic of Iran, Tehran, Iran; 10 Student Research Committee, Department of Health in Disasters and Emergencies, Health Human Resources Research Center, School of Management and Medical Informatics, Shiraz University of Medical Sciences, Shiraz, Iran; 11 Health Policy Research Center, Institute of Health, Shiraz University of Medical Sciences, Fars, Iran

## ⁯

***Dear Editor,***

The first case of the novel coronavirus was reported in Wuhan, Hubei Province, China in December 2019. The disease caused by the virus is called Coronavirus Disease 2019 (COVID-19). Since it is highly contagious and has widely spread throughout the world, the World Health Organization (WHO) declared the pandemic state on 11 March 2020 (Huang et al., 2020[1]). The virus is more contagious compared to Severe Acute Respiratory Syndrome (SARS) and Middle East Respiratory Syndrome (MERS) and can be transmitted via person-to-person contact as well as direct contact with respiratory droplets (Zhu et al., 2020[8]). In addition to the known clinical presentation and consequences of the disease, it has had several profound impacts on the general public’s mental health, and people have shown varying degrees of psychological problems, such as fear and anxiety (Huang et al., 2020[2]; Luo et al., 2020[4]). Another negative aspect of COVID-19 is the social stigma towards the affected ones. This can make people hide their disease, which may interfere with health service provision. Therefore, the virus can spread more and more. Thus, it seems that the psychological effects of COVID-19 and strategies for preventing them should be addressed more in the literature.

After the pandemic declaration, most of the countries implemented different types of mitigation strategies, such as self-isolation, social distancing, mass quarantine, and city lockdown. During these conditions, infected individuals as well as healthy ones have been exposed to short- and long-term psychological consequences. Death of a COVID-19 patient in quarantine conditions and burial by special equipment deprives the family members of their traditional grief and leads to complicated grief syndrome. Quarantine conditions also impose stress, anxiety, irritability, and sleep disorders on the general public (Rajkumar, 2020[5]). Due to the wide range of psychological pressures imposed on societies in economic, political, and social aspects related to COVID-19, increased individual, family, and social violence is expected, as well (WHO, 2020[7]; Usher et al., 2020[6]).

Dissemination of misinformation, misconceptions of people, and the use of negative words are the causes of the social stigma in patients with COVID-19. Individuals who have died, those who have recovered from the disease, and those who have been quarantined as well as their families are exposed to stigma and rejection by the society. The reluctance of friends and relatives to visit the home of a person with COVID-19 due to the fear of spreading the disease to visitors can exacerbate the feelings of isolation, loneliness, and complex emotions. Families and relatives of patients may also experience the feelings of guilt and self-blame due to isolation conditions and inability to care for the sufferers during the pandemic, which can eventually lead to disruption of their daily activities. The mentioned conditions necessitate exceptional psychosocial support for this group of individuals. The adverse psychological effects could be reduced through increasing the general awareness, educating the people about the characteristics of these patients, and having appropriate interaction and empathy among people.

The following solutions can also be useful in reducing the adverse psychological effects:

1.
  1. providing the possibility of transferring the deceased to the city of residence, selection of the burial place by the family, and creating an opportunity for the patient’s relatives to see the body and face of the deceased person in a safe manner,
  2. using clear bags to transport the corpus of the dead one, letting a family member be present during the transfer by ambulance to the burial site, and sharing his or her latest pictures with family members (Huremovic, 2019[3]),
  3. conducting telephone and internet-based consultations in the form of telemedicine for psychological screening,
  4. developing educational startups and applications in this regard,
  5. paying attention to and respecting the local and cultural customs of different societies,
  6. providing spiritual supports in hospitals and health centers,
  7. holding modified debriefing and hot wash sessions using social media for patients and healthy people in quarantine,
  8. providing mental preparedness and adequate explanations about the necessary safety measures for post-quarantine conditions,
  9. strengthening and respecting religious beliefs and supporting them,
  10. holding virtual funerals and ceremonies through the social media,
  11. supporting disadvantaged and vulnerable groups both economically and socially,
  12. providing an agreement to impose unavoidable restrictions on patients and their families,
  13. using relaxation and yoga techniques, and
  14. avoiding following the news.

The following measures can be used to prevent the social stigma:

1. increasing the general population’s knowledge and awareness of the disease,
2. respecting the personality and self-esteem of the infected patients,
3. not using negative words by healthcare providers, people, and the community,
4. producing scientific and educational contents by celebrities, bloggers, social media influencers, and religious leaders,
5. strengthening the cohesion of social ties,
6. sharing the experiences and narratives of COVID-19 patients with others,
7. putting false information and beliefs aside,
8. strengthening the society members’ sense of social responsibility towards each other, and
9. establishing public and multi-organizational information networks to deal with false news about the disease and patients.

## Conclusion

Given that there is no consistent and scientifically validated data on COVID-19, it is common to see the disease as a dangerous and frightening event, which can have adverse psychological effects on people. Challenging the feelings of guilt, processing painful emotions, encouraging grieving people to take care of themselves, and strengthening resilience and the ability to adapt with the consequences of bereavement through psychological support in the form of counseling, psychotherapy, and being in touch with other quarantined families in a safe manner can contribute to people's mental health. The large number of deaths and the need for patients and survivors to receive psychosocial support reflect the necessity of counseling services on a large scale. Hence, reduction of the long-term consequences for families and the society is recommended to be addressed by policymakers and health planners both globally and regionally.

## Notes

Ahmad Soltani and Mahmoudreza Peyravi equally contributed as first authors.

## Acknowledgement

The authors would like to thank Ms. A. Keivanshekouh at the Research Improvement Center of Shiraz University of Medical Sciences for improving the use of English in the manuscript.

## Conflict of interest

The authors have no conflict of interest to declare.

## Funding/Support

Nil.
